# Madrid Nurses’ Perceptions, Needs, and Expectations Regarding Extended Reality-Based Resilience Training: A Multicenter Qualitative Study

**DOI:** 10.3390/healthcare14213763

**Published:** 2026-11-04

**Authors:** Víctor Macho-Foronda, Inés Constanze Hammel, Dania Rocío Díaz-Rodríguez, Cristina Ruiz-Martín, Cristina Veiras-Lorenzo, XRESILIENCE Consortium

**Affiliations:** 1 Hospital Universitario Infanta Leonor, Avenida Gran Vía del Este 80, 28031 Madrid, Spain; victor.macho@salud.madrid.org; 2 Fundación para la Investigación e Innovación Biomédica del Hospital Universitario Infanta Leonor y Hospital Universitario del Sureste, Avenida Gran Vía del Este 80, 28031 Madrid, Spain; 3 Hospital Universitario Infanta Sofía, Paseo de Europa 34, 28702 San Sebastián de los Reyes, Spain; inesconstanze.hammel@salud.madrid.org; 4 Fundación para la Investigación e Innovación Biomédica del Hospital Universitario Infanta Sofía y Hospital Universitario del Henares, Paseo de Europa 34, 28702 San Sebastián de los Reyes, Spain; 5 Hospital Universitario del Henares, Avendida Marie Curie s/n, 28822 Coslada, Spain; 6 Servicio de Urgencias Médicas de Madrid (SUMMA.112), Calle Antracita 2 bis, 28045 Madrid, Spain; c.ruiz@salud.madrid.org (C.R.-M.); cristina.veiras@salud.madrid.org (C.V.-L.); 7 Fundación para la Investigación e Innovación Biosanitaria de Atención Primaria, Avenida Reina Victoria 21, 28003 Madrid, Spain; 8 Division of Psychiatry and Psychotherapeutic Medicine, Medical University of Graz, Auenbruggerplatz 31, 8036 Graz, Austria; info@xr2esilience.eu

**Keywords:** resilience, extended reality, nursing, qualitative research, occupational stress, emotional training, healthcare education

## Abstract

Background: Nurses routinely work under substantial emotional and organizational demands that may challenge their professional well-being and capacity to respond to stressful clinical situations. Resilience training may support healthcare professionals in managing these demands, while Extended Reality (XR) offers opportunities to recreate emotionally and clinically relevant situations within controlled learning environments. Objectives: To explore nurses’ perceptions, needs, and expectations regarding XR-based resilience training and to identify the organizational, professional, and technological conditions that may shape its future implementation. Methods: A multicenter qualitative descriptive-interpretive study was conducted between February and March 2025 across three hospitals and one out-of-hospital emergency medical service in Madrid, Spain. Twenty-six nurses participated in four focus groups. Following open recruitment, participants were purposively selected according to professional profiles to obtain variation in professional experience and clinical settings. Data were analyzed inductively using open coding, constant comparison, progressive category development, analytic memos, and examination of relationships among categories. These procedures were informed by Grounded Theory analytical strategies but were not used to claim a full Grounded Theory methodology. Three researchers independently participated in qualitative coding, and interpretive differences were discussed through consensus. A researcher-developed AI-assisted qualitative analysis tool was used as a complementary resource for corpus exploration, while category development and conceptual interpretation remained researcher-led. Results: Three interrelated categories were identified: expectations regarding XR, perceived barriers, and facilitators and opportunities. Their relationships converged in an interpretive pattern of conditioned openness toward XR. Participants perceived immersive resilience training as potentially valuable when it was realistic, clinically relevant, and provided a safe environment for experiential learning and reflection. However, willingness to engage with XR was constrained by workload, limited training time, technological concerns, and organizational conditions. Protected training time, institutional support, supportive leadership, technical assistance, and professional participation in scenario design emerged as enabling conditions. Conclusions: Participants perceived XR as a potentially useful approach to resilience training, but their openness to its implementation was conditional on clinical relevance and organizational feasibility. The findings do not establish the effectiveness of XR in improving resilience. Rather, they identify professional, organizational, and technological conditions that should inform the participatory design and future evaluation of XR-based resilience interventions.

## 1. Introduction 

Nursing professionals routinely encounter substantial emotional, cognitive, and organizational demands. High workloads, exposure to suffering and clinical uncertainty, responsibility for patient safety, and limited opportunities for recovery may contribute to occupational stress, emotional exhaustion, and burnout [1,2,3,4]. These occupational pressures are relevant not only to nurses’ well-being but also to quality of care, patient safety, and organizational outcomes [3,4].

Professional resilience should not be understood simply as an individual capacity to tolerate adverse working conditions. Rather, it can be conceptualized as a dynamic process arising from the interplay between individual resources and the environmental, relational, and organizational resources available to professionals when responding and adapting to occupational adversity [2,5,6,7]. From this perspective, resilience is not a fixed individual trait but a context-dependent process that may vary according to both personal resources and the characteristics of the work environment. In nursing, professional resilience should therefore be understood in relation to the interaction between individual capacities and contextual conditions, including interpersonal support, team relationships, leadership, organizational resources, and working conditions [2,8].

Resilience is related to, but conceptually distinct from, coping, emotional regulation, well-being, and burnout. These constructs represent different aspects of professionals’ responses to occupational adversity and should not be used interchangeably. In particular, burnout represents an adverse occupational outcome associated with sustained workplace stress, whereas resilience concerns resources and processes involved in adaptation to adversity [2,4,5,6,7].

This distinction is particularly relevant in nursing because interventions intended to strengthen individual resilience may risk shifting attention away from organizational conditions contributing to occupational stress. Resilience-oriented interventions should therefore complement, rather than substitute for, organizational strategies addressing workload, staffing, leadership, workplace support, and other structural determinants of professional well-being [3,4,8].

Digital approaches have increasingly been investigated as potential modalities for resilience and stress-management interventions. App-based and other digital resilience interventions have reported potentially beneficial effects on stress regulation and mental health, while systematic-review evidence supports continued evaluation of digitally delivered resilience training rather than assuming uniform effectiveness across professional populations and contexts [5,6].

Simulation and immersive technologies provide additional opportunities for experiential learning in healthcare. Extended Reality (XR), encompassing virtual, augmented, and mixed-reality environments, can provide controlled settings in which healthcare professionals engage with simulated clinical situations and rehearse responses without exposing patients to the consequences of real-world errors [9,10,11,12,13]. Recent evidence concerning XR and computer-based simulation in health professions education supports the educational relevance of immersive and contextually realistic simulation while also emphasizing the importance of appropriate instructional and scenario design [13].

Immersive technologies have also been investigated in relation to stress management, mindfulness, burnout, work engagement, emotional experience, and mental health training [7,9,10,11,12,14,15]. Initiatives such as resilience training for nurses using XR glasses have also been developed [16]. Studies involving healthcare professionals have reported potentially beneficial experiences with VR-based relaxation, mindfulness, and educational interventions [7,9,15]. Nevertheless, this evidence is heterogeneous and does not establish the effectiveness of XR-based resilience training specifically.

The feasibility of an educational technology cannot, however, be inferred solely from its technical capabilities or from outcomes observed under controlled study conditions. Implementation in healthcare also depends on perceived clinical relevance, usability, compatibility with existing workflows, available resources, and the conditions under which professionals are expected to engage with training [12,13,17]. A technology perceived as innovative or educationally promising may therefore have limited acceptability if participation adds further demands within an already pressured clinical environment [3,4,8].

Although immersive technologies are increasingly being investigated in healthcare education and in interventions related to stress and professional well-being [7,9,10,11,12,13,14,15,16,18], comparatively less is known about how bedside nurses perceive the acceptability, clinical relevance, and organizational feasibility of using XR specifically for resilience training before implementation. In particular, limited attention has been given to how working conditions, emotional demands, technological expectations, and organizational support interact in shaping willingness to engage with such training.

A qualitative approach is appropriate for addressing this gap because it enables investigation of participants’ perspectives and the meanings and contextual conditions underlying their evaluations rather than reducing acceptability to predetermined variables. A qualitative descriptive orientation is particularly suitable for applied health research when the aim is to provide a comprehensive account of participants’ perspectives while remaining close to their experiences and language [19]. Therefore, this study aimed to explore nurses’ perceptions, needs, and expectations regarding XR-based resilience training and to identify the professional, organizational, and technological conditions that may shape its future implementation across different healthcare settings in Madrid.

## 2. Materials and Methods

### 2.1. Study Design

This study was conducted within the framework of the XR^2^ESILIENCE project [20]. A multicenter qualitative descriptive-interpretive study was conducted to explore nurses’ perceptions, needs, and expectations regarding resilience training through Extended Reality (XR). A qualitative descriptive orientation was selected because it supports a comprehensive account of participants’ perspectives while remaining relatively close to their experiences and language, with interpretive development appropriate to applied health research [19].

The analytical process was inductive and informed by selected procedures associated with Grounded Theory, particularly open coding, constant comparison, progressive category development, memo writing, and examination of relationships among categories [21]. These procedures were used as analytical strategies rather than as a claim to a full Grounded Theory methodology. Data collection was completed before the full analytical process, and recruitment was not iteratively redirected in response to an emerging theory; consequently, theoretical sampling and theoretical saturation are not claimed. Qualitative data were managed and coded using ATLAS.ti (version 25; ATLAS.ti Scientific Software Development GmbH, Berlin, Germany).

The Standards for Reporting Qualitative Research (SRQR) were used to guide transparent reporting of the study [22].

### 2.2. Context and Participants

The study was conducted between February and March 2025 across four healthcare organizations in the Madrid region, Spain: three hospital settings and the regional out-of-hospital emergency medical service. A total of 26 nurses involved in direct clinical care participated in four focus groups, one in each participating organization.

Recruitment was initially open and was facilitated through a QR-code invitation available to potentially eligible nursing professionals. Volunteers meeting the eligibility criteria were subsequently selected purposively according to professional profiles relevant to the research question. Selection sought variation in professional experience and clinical context.

Participants represented a broad range of professional nursing experience, from 5 to 33 years (median, 15.5 years). To provide information relevant to transferability while avoiding overly granular participant profiles, professional experience was summarized in broad categories: 7 participants (26.9%) had less than 10 years of experience, 9 (34.6%) had 10–19 years, and 10 (38.5%) had 20 years or more. Participants were engaged in direct clinical care across a range of practice environments, including critical care, hospital emergency care, general inpatient care, mental health, and out-of-hospital emergency care.

Inclusion criteria were active employment as a nursing professional within one of the participating healthcare organizations, involvement in direct clinical care, and willingness to discuss experiences related to occupational stress, emotional coping, professional resilience, and expectations regarding XR-based training. Participants were informed that the research formed part of the XR^2^ESILIENCE project. No financial incentives were provided.

Professional characteristics are intentionally reported at an aggregated level. Because the focus groups were conducted within participants’ own healthcare organizations and some professional profiles were represented by small numbers of nurses, combinations of workplace, specific clinical unit, years of professional experience, shift pattern, employment characteristics, and other individual attributes could function as quasi-identifiers and increase the risk of deductive disclosure. Accordingly, participant-level combinations of these characteristics are not reported. This approach was adopted to provide sufficient contextual information for assessing transferability while preserving participant confidentiality (Table 1).

### 2.3. Data Collection

Focus groups were selected to facilitate interaction among participants and to explore shared and divergent perspectives concerning occupational stress, resilience, and the potential use of XR for resilience training. The focus-group procedure was informed by established methodological guidance for applied focus-group research [23]. Four focus groups were conducted, each lasting approximately 90 min.

The focus groups were facilitated by two external professionals with expertise in focus-group research, who fulfilled moderator and observer functions. They were external to the XR^2^ESILIENCE research team responsible for the subsequent qualitative analysis. Participants were informed of the purpose of the research and its association with XR^2^ESILIENCE.

Sessions were audio- and video-recorded with explicit consent. Audio recordings were transcribed into text, and video recordings were subsequently reviewed to enrich the transcripts with relevant contextual and interactional information. Transcripts were anonymized before analysis.

The discussion guide addressed four broad areas: (1) experiences of occupational stress and emotional coping; (2) perceptions of resilience in professional nursing practice; (3) expectations, perceived usefulness, and concerns regarding XR-based resilience training; and (4) personal, technological, and organizational conditions that could facilitate or hinder implementation.

### 2.4. Data Analysis

**Figure 1:**
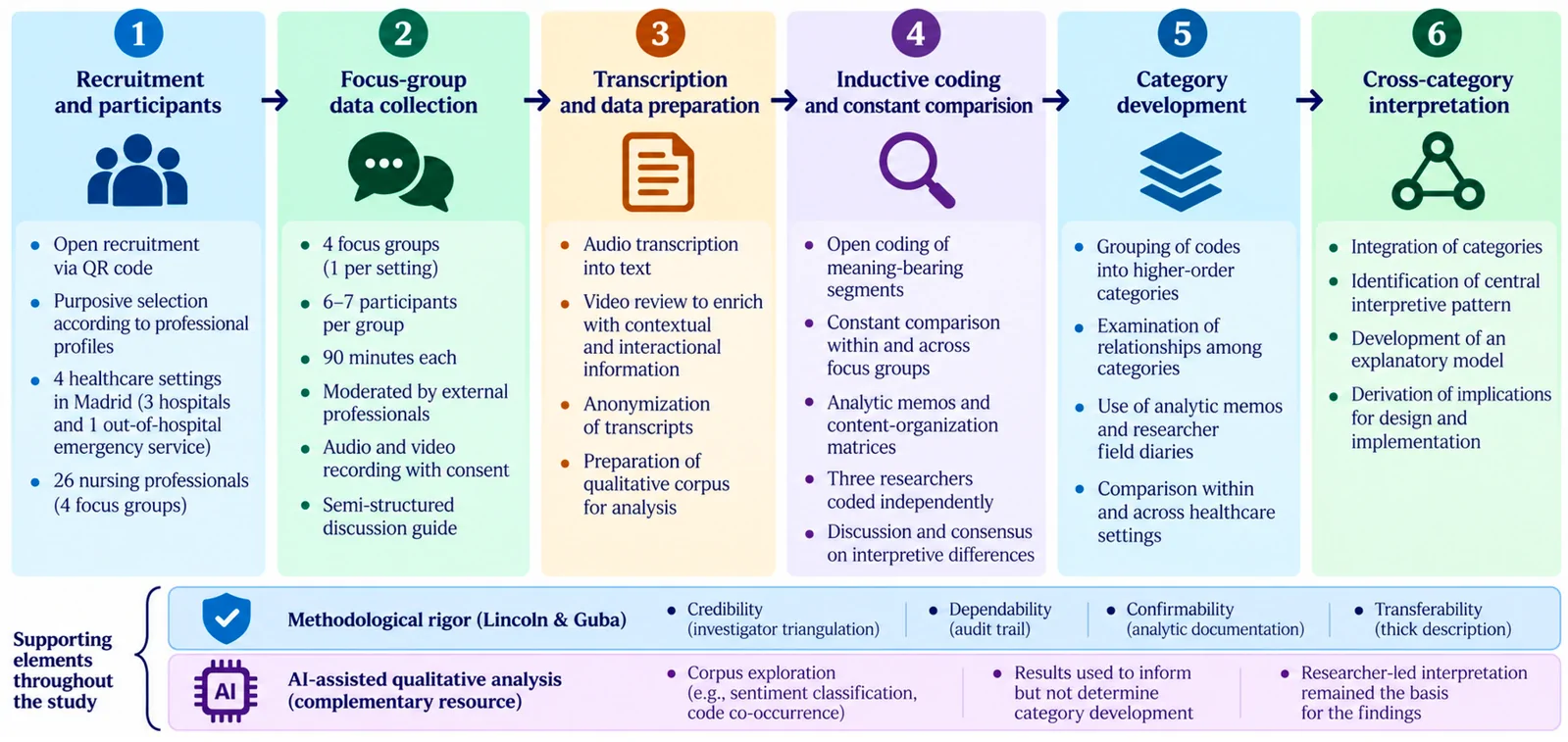
Study design and analytical process.

Analysis began after completion and transcription of all four focus groups. The researcher-led qualitative analysis followed an inductive and constant-comparative process informed by selected Grounded Theory analytical procedures [21]. Transcripts were first examined through open coding to identify meaning-bearing textual segments relevant to occupational stress, resilience, perceptions of XR, implementation concerns, and proposed enabling conditions (the study design and analytical process are summarized in Figure 1).

Codes were compared within and across focus groups and progressively organized into higher-order categories. Constant comparison was used to examine similarities, differences, and relationships across coded material [21]. Relationships among categories were subsequently examined to move from descriptive classification toward an interpretive understanding of the conditions shaping participants’ openness to XR-based resilience training.

Three researchers from the XR^2^ESILIENCE research team participated independently in qualitative coding. Independent interpretations were subsequently compared, and differences in coding or category interpretation were discussed in consensus meetings. Investigator triangulation was used to examine alternative interpretations rather than to calculate statistical inter-rater agreement.

Analytic memos, researcher field diaries, and content-organization matrices were used to document category development, emerging relationships, analytical decisions, and changes in interpretation. Comparisons were conducted both within individual focus groups and across the four healthcare contexts.

A researcher-developed AI-assisted qualitative analysis tool was additionally used as a complementary resource for corpus exploration, including sentiment classification and examination of code co-occurrences. These computational outputs were analytically distinguished from researcher-led coding and interpretive development of categories. They were not treated as independent qualitative findings or as substitutes for researcher interpretation.

Sentiment classification was used descriptively to explore positive, negative, and neutral orientations in the textual corpus. Because the existing analytical outputs use different levels of textual segmentation, sentiment frequencies were not used to infer prevalence, representativeness, or conceptual importance. The primary findings reported in this article are therefore based on researcher-led qualitative interpretation rather than numerical sentiment frequencies.

### 2.5. Methodological Rigor

Trustworthiness was addressed according to the criteria of credibility, dependability, confirmability, and transferability described by Lincoln and Guba [24]. Credibility was supported through investigator triangulation, with three researchers independently engaging in coding and subsequently comparing interpretations in consensus discussions.

Dependability and confirmability were supported through documentation of analytical decisions using analytic memos, researcher field diaries, and content-organization matrices, providing an audit trail of category development and interpretive decisions [24]. Interpretations and higher-order categories were repeatedly examined against supporting transcript material during the analytical process.

Comparison across four healthcare settings was used to identify recurrent patterns and context-related differences rather than assuming homogeneity across settings. Transferability was supported through description of the clinical contexts, recruitment process, and professional profiles sought [24]. The absence of detailed participant-level sociodemographic information nevertheless limits assessment of transferability according to individual characteristics.

### 2.6. Research Team and Reflexivity

Members of the analytical research team were involved in the broader XR^2^ESILIENCE project and therefore approached the study with prior professional knowledge of its objectives and the potential application of immersive technologies to resilience training. This positioning was considered during interpretation because involvement in the project could shape expectations regarding the usefulness or feasibility of XR.

To provide separation between project development and immediate data generation, focus groups were moderated and observed by external professionals experienced in focus-group research. Participants were informed of the study’s association with XR^2^ESILIENCE. During analysis, independent coding, consensus discussions, analytic memos, researcher field diaries, content-organization matrices, and comparison with source material were used to make interpretive decisions explicit and examine alternative readings of the data.

### 2.7. Ethical Considerations

The study was approved by the Ethics Committee for Research with Medicinal Products (CEIm) of Hospital Universitario de Getafe on 30 January 2025 (CEIm24/125) and was conducted in accordance with the Declaration of Helsinki. All participants provided written informed consent. Data were anonymized and securely stored in accordance with Regulation (EU) 2016/679 and Spanish Organic Law 3/2018 of 5 December on Personal Data Protection and guarantee of digital rights. No financial incentives were provided for participation.

### 2.8. Use of Artificial Intelligence

A distinction was made between AI-assisted analytical support and generative AI used during manuscript preparation. As described in Section 2.4, a researcher-developed AI-assisted qualitative analysis tool was used as a complementary resource for corpus exploration. Researcher-led coding and interpretation remained the basis for category development and the conceptual findings reported in the study.

During preparation of the manuscript, Claude AI (Claude Opus 4.7; Anthropic, San Francisco, CA, USA), ChatGPT (GPT-5.5; OpenAI, San Francisco, CA, USA), and Microsoft Copilot (Microsoft Corporation, Redmond, WA, USA). The tools were used in their paid versions for text drafting, language refinement, and editorial support. These general-purpose generative AI tools were not used as autonomous decision-makers in the interpretation of the qualitative findings. All manuscript content and interpretations were critically reviewed by the authors, who take responsibility for the final content. 

## 3. Results

Analysis of the four focus groups identified three interrelated categories concerning nurses’ perceptions of XR-based resilience training: (1) expectations regarding XR; (2) perceived barriers; and (3) facilitators and opportunities (Table 2)**.** Rather than functioning as independent domains, these categories interacted around a broader interpretive pattern: participants expressed openness toward XR, but this openness was conditional on whether the technology addressed clinically meaningful needs without adding further burden to already demanding working conditions (Table 2) and Illustrative participant quotations Table 3.

Comparison across the four healthcare contexts identified recurrent concerns regarding workload, emotional demands, training feasibility, technological usability, and organizational support. Supplementary computational analyses were used only for corpus exploration and did not determine the conceptual importance of the categories.

### 3.1. Expectations Regarding XR: Clinical Realism and Safe Experiential Learning

Participants generally expressed interest in XR-based resilience training. Immersive environments were perceived as potentially useful when they reproduced recognizable clinical and emotional situations and allowed participants to rehearse responses without the consequences associated with errors in real clinical practice.

*“I would like the scenarios to be as realistic as possible, so we can prepare for common situations, like when a patient deteriorates, or a family reacts badly.” (FG2-P5)*

*“I think it could help us notice how we respond under pressure, without having to wait to experience it during a real shift.” (FG3-P2)*

*“In a virtual environment you can make mistakes, get emotional or freeze, but then you reflect and learn; at work that’s not always possible.” (FG1-P4)*

These accounts suggest that participants did not value XR primarily because of technological novelty. Its perceived value was linked to the possibility of recreating emotionally recognizable clinical situations while creating a protected opportunity for experiential learning and reflection. Realism therefore functioned as a condition for perceived educational relevance rather than simply as a technological preference.

### 3.2. Perceived Barriers: Structural Pressure Constrains Engagement

Despite general interest, participants identified barriers that could limit implementation. Heavy workload, limited time for professional development, technological complexity, and previous difficulties with digital tools generated pragmatic concerns regarding feasibility.

*“We are very eager to try it, but sometimes there’s barely time to breathe during a shift.” (FG1-P5)*

*“We would like to receive this training, but if it requires a lot of time or complicated equipment, it will be difficult to sustain.” (FG4-P3)*

*“We already have many digital tools, and they don’t always work; sometimes technology makes things more complicated.” (FG2-P6)*

These concerns were directed less toward XR as an abstract technology than toward the possibility that training could become another demand within an already pressured working environment. Technological acceptance was therefore intertwined with organizational feasibility. Interest in innovation did not eliminate concerns about time, workload, or whether technology would simplify or complicate clinical work.

### 3.3. Facilitators and Opportunities: From Interest to Feasible Participation

Participants identified several conditions that could facilitate adoption, including motivation to improve emotional coping, institutional support, protected training time, technical assistance, and participation of nurses in the development of scenarios.

*“We really need this type of training, because many times we don’t have space to talk about how we feel.” (FG3-P7)*

*“If management supports it and it can be integrated into working hours, it would be very positive.” (FG4-P2)*

*“It would be important for us to help design the scenarios ourselves, so they resemble what we experience every day.” (FG1-P2)*

These facilitators represented more than practical implementation resources. Protected time and organizational support signaled recognition of nurses’ educational and emotional needs, while co-design connected technological development with professional ownership and clinical authenticity. Participants therefore appeared more willing to envisage using XR when its implementation involved nurses rather than positioning them merely as end users.

### 3.4. Cross-Category Interpretation: Conditioned Openness Toward XR

Cross-category comparison identified conditioned openness toward XR as the central interpretive pattern connecting expectations, barriers, and facilitators. Participants were generally interested in XR-based resilience training, but willingness to engage with it was neither unconditional nor determined solely by attitudes toward technology.

Substantial emotional demands, workload pressure, and limited opportunities for structured reflection contributed to a perceived need for training capable of supporting emotional coping and preparation for difficult clinical situations. XR was perceived positively when participants envisaged realistic scenarios, safe opportunities to rehearse responses, and opportunities for subsequent reflection.

At the same time, the organizational environment that generated the perceived need for resilience training could constrain participation in that training. Heavy workload, lack of protected learning time, technological complexity, and negative experiences with digital systems could transform initial interest into pragmatic resistance if XR were experienced as an additional burden.

Conversely, protected training time, organizational support, accessible technology, supportive leadership, technical assistance, and professional participation in scenario design increased perceived feasibility and relevance.

**Figure 2:**
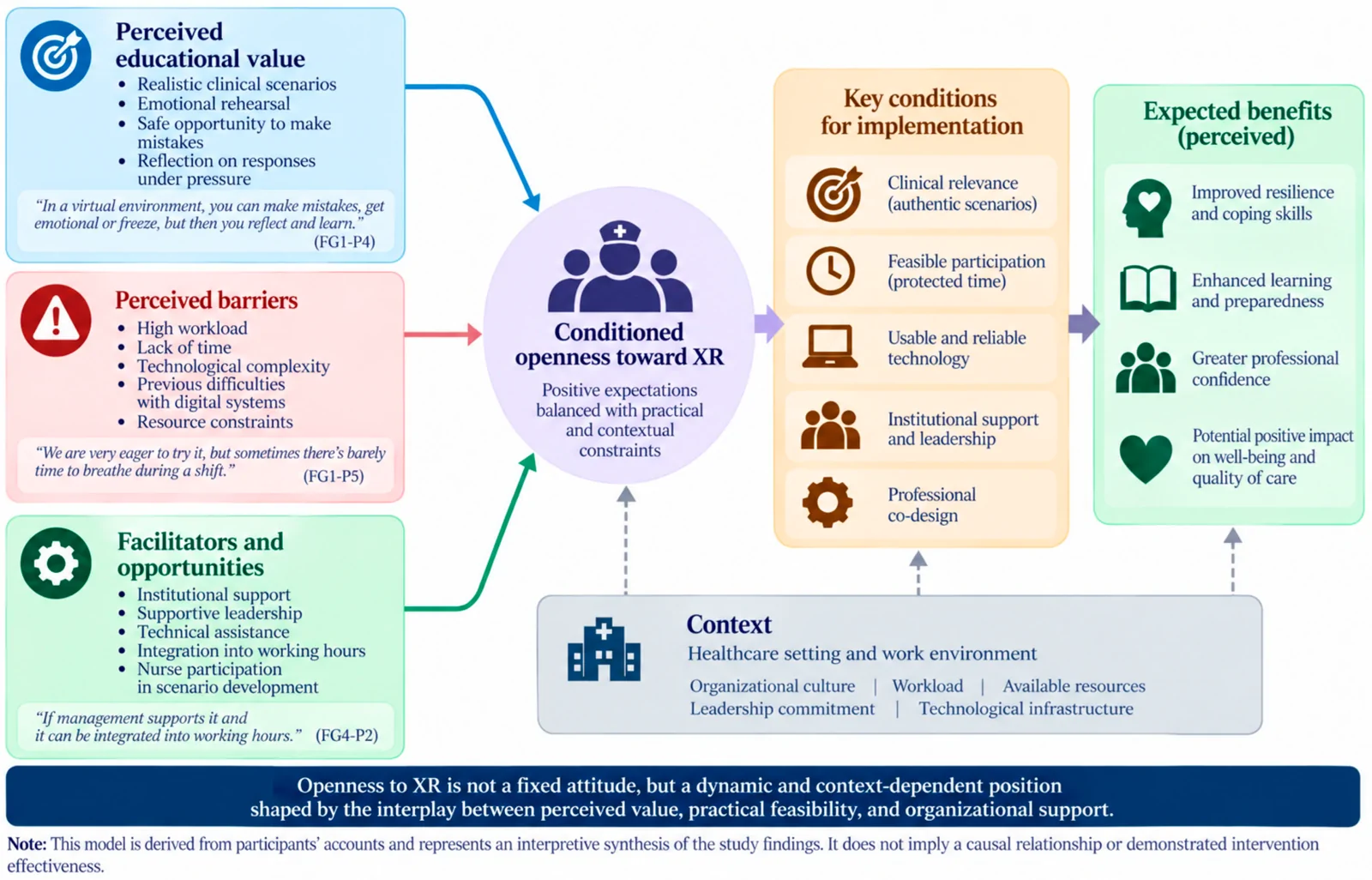
Interpretive model of conditioned openness toward XR-based resilience training.

Conditioned openness therefore represents a dynamic relationship rather than a stable attitude toward XR. Greater alignment among clinical relevance, organizational support, usability, and feasible participation strengthened perceived acceptability; when these conditions were absent, positive expectations were tempered by pragmatic concerns (Figure 2). The design and implementation considerations derived from these findings are presented in Table 4.

## 4. Discussion

This study identified conditioned openness toward XR as the principal interpretive pattern connecting nurses’ expectations, perceived barriers, and enabling conditions. Participants perceived potential value in XR when it offered realistic and safe experiential learning but simultaneously evaluated whether participation would be feasible within their actual working environment.

### 4.1. Conditioned Openness and Organizational Feasibility

The context-dependent acceptability identified in this study provides an important qualification to the generally promising results reported for digital resilience and immersive interventions. Digital resilience training has demonstrated potential benefits across different populations and delivery formats [5,6], while VR-based interventions involving healthcare professionals have reported promising findings in relation to stress regulation, mindfulness, burnout, work engagement, and emotional experience [7,9,15]. Nevertheless, these studies do not establish that an intervention that is effective or acceptable under study conditions will necessarily be feasible within routine nursing workflows.

The present findings indicate that workload, available time, technical reliability, clinical relevance, and institutional support form part of nurses’ appraisal of XR. This interpretation is consistent with literature showing that nurses’ psycho-emotional status, burnout, and professional outcomes are strongly influenced by working conditions and organizational pressures [2,3,4,8]. Thus, reluctance toward XR should not necessarily be interpreted as resistance to technological innovation; in this study, it frequently represented a pragmatic assessment of implementation feasibility.

A particularly relevant finding is that the organizational pressures generating a perceived need for resilience support may simultaneously restrict professionals’ ability to participate in resilience training. This reinforces the need to position resilience interventions alongside, rather than in place of, organizational approaches addressing workload, staffing, leadership, and professional well-being [3,4,8].

### 4.2. Resilience as a Relational and Organizational Process

Participants’ accounts suggest that resilience training should not be framed exclusively as an individual intervention. Opportunities for reflection, team support, supportive leadership, professional recognition, and organizational conditions were intertwined with perceptions of coping and resilience. This interpretation is consistent with evidence indicating that resilience among healthcare professionals is associated with contextual and occupational conditions as well as individual resources [2,8,18].

This finding is important because resilience interventions can otherwise risk positioning professionals as individually responsible for adapting to working conditions that are partly organizational in origin. Evidence linking nursing workload and burnout with adverse professional, organizational, and patient outcomes reinforces the need to avoid this reduction [3,4]. XR-based resilience training should therefore be considered a potential educational resource within a broader organizational strategy rather than a technological solution to structurally generated occupational stress.

### 4.3. Experiential Learning, Realism, and Co-Design

Clinical and emotional realism emerged as a central expectation. Participants wanted scenarios that resembled situations encountered in practice and that allowed emotional responses to be explored safely. This finding is consistent with the educational rationale underlying immersive simulation, in which contextual authenticity and active engagement contribute to experiential learning [12,13,25].

Recent evidence concerning XR and computer-based simulation in health professions education indicates that immersive environments can support clinically relevant learning processes, although educational value depends on scenario design, learning objectives, and integration of the technology into an appropriate educational process [12,13,25]. The present findings extend this consideration to resilience-oriented training: participants’ interest was directed less toward immersion itself than toward whether immersive scenarios represented emotionally recognizable professional situations.

The value participants attributed to safe rehearsal and reflection is also compatible with previous research examining VR-based stress-management, mindfulness, relaxation, and educational interventions among healthcare professionals [7,9,15]. However, because participants in the present study had not completed the proposed XR resilience intervention, these convergences should be interpreted as supporting the plausibility of their expectations rather than demonstrating an intervention effect.

Co-design may be particularly important in this context. Participants’ desire to contribute to scenario development suggests that professional participation could improve perceived authenticity, relevance, and ownership. Within the present study, co-design therefore emerged not merely as a technical development strategy but as a potential implementation mechanism connecting technological design with nurses’ experiential knowledge.

### 4.4. Implications for Implementation and Future Research

The findings identify several considerations for future development and evaluation of XR-based resilience interventions. Training should be integrated into existing work structures rather than added as an extracurricular demand, particularly given the documented relationship between nursing workload, occupational stress, burnout, and professional outcomes [2,3,4,8]. Protected participation time, accessible technical requirements, and appropriate organizational support should therefore be considered during implementation planning.

Future development should also preserve opportunities for structured reflection and debriefing. The literature on immersive simulation indicates that the educational value of XR depends not simply on technological immersion but also on instructional design and integration within a meaningful educational process [12,13,25]. For resilience-oriented XR, reflection on emotional and behavioral responses may therefore be as important as exposure to the simulated event itself.

These findings concern anticipated implementation rather than effectiveness. Existing studies of digital resilience interventions and VR-based approaches provide a rationale for further evaluation [5,6,7,9,15], but the present study cannot determine whether XR exposure improves resilience, reduces stress, or changes occupational outcomes. Future studies should therefore evaluate actual XR exposure and examine usability, post-exposure acceptability, learning processes, and resilience or occupational well-being outcomes using designs appropriate to those questions.

### 4.5. Strengths and Limitations

A strength of this study is the inclusion of four healthcare contexts, including hospital and out-of-hospital emergency settings. Recruitment sought variation in professional profiles and included nurses from clinical environments with differing demands. Focus groups were facilitated by external professionals experienced in group-based qualitative research, providing separation between the XR^2^ESILIENCE analytical team and immediate data generation. Independent coding by three researchers, consensus discussions, analytic memos, researcher field diaries, and content-organization matrices provided additional transparency and traceability to the analytical process.

Several limitations should nevertheless be considered. Recruitment was voluntary and subsequently purposive, creating potential self-selection bias. Professionals interested in education, resilience, or technological innovation may have been more willing to participate. Furthermore, participants were recruited from organizations participating in XR^2^ESILIENCE, and awareness of the project context may have influenced expectations regarding XR.

Detailed participant-level sociodemographic information was not systematically collected. Consequently, the findings cannot support robust comparisons according to age, gender, or other individual characteristics. This also limits assessment of transferability to populations differentiated by these variables.

All four focus groups were completed before the full analytical process. Consequently, recruitment could not be iteratively redirected in response to emerging categories, and the study does not claim theoretical sampling or theoretical saturation.

Participants discussed expectations and perceptions of XR-based resilience training rather than evaluating a completed XR intervention. Findings therefore concern anticipated acceptability and implementation conditions and cannot establish effectiveness, usability after actual exposure, or effects on resilience, stress, burnout, or clinical outcomes.

Finally, an AI-assisted analytical tool was used as a complementary resource for corpus exploration. Because computational classification may introduce segmentation or classification errors, computational outputs were treated as supplementary and did not determine researcher-developed categories or conceptual interpretation. Numerical sentiment outputs were not interpreted as prevalence estimates.

## 5. Conclusions

This multicenter qualitative study identified a pattern of conditioned openness toward XR-based resilience training among nurses participating across hospital and out-of-hospital emergency settings in Madrid.

Participants perceived immersive training as potentially valuable when it could reproduce clinically and emotionally meaningful situations, provide opportunities for safe experiential learning, and facilitate reflection on responses to stressful events. However, openness was strongly shaped by organizational feasibility. Workload, limited training time, technological complexity, and resource constraints could temper initial interest, whereas protected learning time, supportive leadership, technical assistance, and professional involvement in scenario design increased perceived acceptability.

These findings should not be interpreted as evidence that XR improves resilience or reduces occupational stress because no XR intervention was evaluated. Rather, they identify professional, organizational, and technological conditions that should be considered when designing and subsequently evaluating XR-based resilience interventions. Participatory co-design and integration of training within existing organizational structures may be particularly important for ensuring that future interventions respond to nurses’ clinical and emotional realities.

## Figures and Tables

**Table 1:** Study settings, recruitment, and focus-group composition.

Characteristic	Description/Category	*n* (%)
Total participants	Nurses	26 (100)
Focus groups	Focus groups conducted	4
Healthcare context	Hospital settings	20 (76.9)
	Out-of-hospital emergency medical service	6 (23.1)
Professional role	Nurses involved in direct clinical care	26 (100)
Professional experience	<10 years	7 (26.9)
	10–19 years	9 (34.6)
	≥20 years	10 (38.5)
Professional experience, range	5–33 years	—
Professional experience, median	15.5 years	—
Clinical practice environments represented	Critical care; hospital emergency care; general inpatient care; mental health; out-of-hospital emergency care	—

**Table 2:** Analytical Categories and Their Contribution to Conditioned Openness Toward XR.

Analytical Category	What Participants Emphasized	Interpretive Meaning	Implications for XR Design and Implementation
Expectations regarding XR	Realistic clinical situations; emotional rehearsal; safe opportunities to make mistakes; reflection on responses under pressure	Perceived educational value depended primarily on clinical and emotional authenticity rather than technological novelty	Develop clinically realistic and emotionally meaningful nursing scenarios and incorporate opportunities for reflection
Perceived barriers	Heavy workload; limited training time; technological complexity; previous difficulties with digital tools; resource constraints	Structural and organizational pressures constrained engagement; initial interest could be reduced if XR training was experienced as an additional workload demand	Provide protected training time, accessible and reliable technology, adequate resources, and technical support
Facilitators and opportunities	Institutional support; supportive leadership; technical assistance; integration into working hours; nurses’ participation in scenario development	Organizational support could translate initial interest into feasible participation, while professional involvement could strengthen perceived relevance and ownership	Integrate training into existing work structures and involve nurses in participatory scenario design
Cross-category interpretation	Perceived educational value was weighed against organizational and technological feasibility	Openness toward XR was conditional and context-dependent rather than a stable attitude toward the technology	Align clinical relevance, usability, organizational support, and feasible participation when designing and implementing XR-based resilience training

**Table 3:** From Empirical Accounts to Cross-Category Interpretation.

Analytical Dimension	Illustrative Participant Account	Interpretive Reading	Contribution to Cross-Category Interpretation
Clinical realism	“I would like the scenarios to be as realistic as possible, so we can prepare for common situations, like when a patient deteriorates or a family reacts badly.” (FG2-P5)	Clinical and emotional authenticity shaped the perceived relevance of XR-based training	Perceived educational value
Safe experiential learning	“In a virtual environment you can make mistakes, get emotional or freeze, but then you reflect and learn; at work that’s not always possible.” (FG1-P4)	XR was valued as a potentially protected environment for experiential learning, emotional rehearsal, and reflection	Perceived educational value
Workload pressure	“We are very eager to try it, but sometimes there’s barely time to breathe during a shift.” (FG1-P5)	Interest in XR coexisted with concerns about whether participation would be feasible under existing workload pressures	Implementation constraints
Implementation burden	“We would like to receive this training, but if it requires a lot of time or complicated equipment, it will be difficult to sustain.” (FG4-P3)	Time requirements and technological complexity could transform an educational opportunity into an additional work demand	Implementation constraints
Organizational support	“If management supports it and it can be integrated into working hours, it would be very positive.” (FG4-P2)	Institutional support and integration into working time increased the perceived feasibility of participation	Enabling conditions
Professional co-design	“It would be important for us to help design the scenarios ourselves, so they resemble what we experience every day.” (FG1-P2)	Professional participation connected scenario authenticity with relevance and ownership	Enabling conditions
Cross-category integration	No single quotation was used to represent this higher-order interpretation; it was developed through comparison of patterns across the four focus groups	Perceived educational value was continuously appraised in relation to organizational and technological feasibility	Conditioned openness toward XR

**Table 4:** Implementation Framework Derived from Nurses’ Perceptions of XR-Based Resilience Training.

Implementation Domain	Need or Concern Identified in Participants’ Accounts	Design or Implementation Consideration	Intended Implementation Function
Clinical relevance	Training should reflect recognizable clinical situations and professional challenges	Develop scenarios grounded in realistic nursing situations and contextually relevant stressors	Strengthen perceived relevance and applicability of XR-based training
Emotional learning	Participants valued opportunities to experience, recognize, and reflect on emotional responses in a safe environment	Include emotionally meaningful scenarios together with structured opportunities for reflection and debriefing	Support experiential learning and reflection on responses to stressful situations
Work organization	Heavy workload and limited time may restrict participation and make training an additional burden	Provide protected training time and integrate participation into existing work structures	Improve feasibility of participation without adding avoidable workload demands
Technology and usability	Technological complexity and previous difficulties with digital systems may reduce willingness to engage	Use accessible, reliable, and manageable XR systems and provide appropriate technical assistance	Reduce practical and technological barriers to participation
Leadership and institutional support	Implementation was perceived as more feasible when supported by management and the organization	Ensure visible organizational commitment, supportive leadership, and adequate implementation resources	Create organizational conditions that enable participation
Professional participation	Nurses expressed a preference for contributing to the development of scenarios	Involve nursing professionals in participatory scenario design and iterative refinement	Strengthen clinical authenticity, relevance, and professional ownership
Evaluation and future research	Participants discussed anticipated value and feasibility rather than experiences of a completed XR resilience intervention	Evaluate actual XR exposure, usability, post-exposure acceptability, learning processes, and appropriate resilience or occupational well-being outcomes	Determine feasibility and outcomes empirically rather than inferring effectiveness from pre-implementation perceptions

## Data Availability

The data presented in this study are not publicly available due to ethical and privacy restrictions. Qualitative data contain information that could compromise participant confidentiality. Anonymized data may be available from the corresponding author upon reasonable request and subject to approval by the relevant Ethics Committee.

## Abbreviations

XR^2^ESILIENCE	Extended Reality for Resilience
SERMAS	Madrid Regional Health Service
SUMMA 112	Medical Emergency Service of Madrid
XR	Extended Reality
SRQR	Standards for Reporting Qualitative Research
AI	Artificial Intelligence
FG	Focus Group
CEIm	Ethics Committee for Research with Medicinal Products
